# Utilization of Computer Classification Methods for Exposure Prediction and Gene Selection in *Daphnia magna* Toxicogenomics

**DOI:** 10.3390/biology12050692

**Published:** 2023-05-09

**Authors:** Berkay Paylar, Martin Längkvist, Jana Jass, Per-Erik Olsson

**Affiliations:** 1 The Life Science Center-Biology, School of Science and Technology, Örebro University, SE-701 82 Örebro, Sweden; 2 Center for Applied Autonomous Sensor Systems, Örebro University, SE-701 82 Örebro, Sweden

**Keywords:** Zn, water hardness, biomarker, bioavailability, machine learning

## Abstract

**Simple Summary:**

The toxicogenomic approach has gained increased attention for understanding key molecular events within organisms to deal with exposure conditions. However, finding relevant biomarkers to predict toxicity still remains a challenging task. In this study, we investigated machine learning applications as a tool to aid toxicity prediction and gene ranking method by training eight different standard supervised machine learning classifiers with qPCR gene expression data. We have shown that the toxicogenomic approach can benefit greatly from machine learning to identify unique expression profiles and predict toxicity, as well as visualization of genes by using Shapley values.

**Abstract:**

Zinc (Zn) is an essential element that influences many cellular functions. Depending on bioavailability, Zn can cause both deficiency and toxicity. Zn bioavailability is influenced by water hardness. Therefore, water quality analysis for health-risk assessment should consider both Zn concentration and water hardness. However, exposure media selection for traditional toxicology tests are set to defined hardness levels and do not represent the diverse water chemistry compositions observed in nature. Moreover, these tests commonly use whole organism endpoints, such as survival and reproduction, which require high numbers of test animals and are labor intensive. Gene expression stands out as a promising alternative to provide insight into molecular events that can be used for risk assessment. In this work, we apply machine learning techniques to classify the Zn concentrations and water hardness from *Daphnia magna* gene expression by using quantitative PCR. A method for gene ranking was explored using techniques from game theory, namely, Shapley values. The results show that standard machine learning classifiers can classify both Zn concentration and water hardness simultaneously, and that Shapley values are a versatile and useful alternative for gene ranking that can provide insight about the importance of individual genes.

## 1. Introduction

The amount of zinc (Zn) intake for humans has an influence for many physiological functions that can cause both deficiency and toxicity symptoms. The recommended dietary allowance (RDA) for the essential trace metal Zn for adults over 19 years old is 11 mg/d for men and 8 mg/d for women [1]. A deficiency of Zn can cause symptoms of nausea, vomiting, epigastric pain, and fatigue, while an excess intake of Zn well above the RDA (100–300 mg/d) can cause Zn toxicity that manifests in induced copper deficiency with symptoms of anemia and neutropenia [2]. Zn is naturally found in food products, where certain foods have a higher level of Zn than others, and drinking water, where the water hardness has a crucial influence on Zn bioavailability. Therefore, estimating both the Zn concentration and the hardness of drinking water is of interest for human health risk assessment. To accurately assess the health risks associated with water quality, it is crucial to consider both the concentration of Zn and the hardness of the water. However, the selection of exposure media for traditional toxicology tests is often limited to predetermined hardness levels, which may not reflect the diverse range of water chemistry compositions observed in natural environments. Furthermore, many traditional toxicology tests rely on whole organism endpoints, such as survival and reproduction, which not only require a large number of test animals but can also be time-consuming and labor-intensive. For instance, OECD guidelines for *Daphnia magna* reproduction test lasts for 21 days [3]. Assessing differences in offspring production by identifying the key biomarker genes and monitoring their expression levels in earlier stages stands out as a promising alternative for risk assessment.

Organisms regulate their gene expression to cope with the changes in water chemistry to maintain homeostasis. Analysis of the key genes that respond to changing hardness and Zn levels can be used to generate representative expression profiles for unique combinations of the two variables. These profiles can be used to predict the exposure conditions of test subjects from different origins. There are various approaches for collecting gene response data for studying toxicological outcomes of organisms, such as quantitative PCR (qPCR), microarrays, and RNA-Seq. qPCR is commonly used to gain insights into emerging toxicity [4]. It is low-cost, simple, and the most accurate method for expression analysis, but it only allows for the testing of a limited number of genes and requires pre-selection of the genes. Microarrays can be used to analyze large numbers of genes with medium cost. However, microarrays have low sensitivity for very lowly or very highly expressed genes as well as high noise. RNA-Seq is a high-cost alternative that has high range and ability to identify novel transcripts. RNA-Seq requires high data processing and storage, and results usually need to be verified by qPCR. Therefore, identifying the key genes that contribute to unique gene expression profiles in different water hardness and Zn concentrations is of interest to aid pre-selection requirement of using qPCR.

Machine learning (ML) is a subfield of artificial intelligence (AI) that can construct hypotheses and find patterns to explain complex relationships in the data and is therefore suitable for molecular biology data. ML has previously been used for water quality assessment [5], microarray gene expression data [6,7,8,9,10], qPCR gene expression [11,12,13], and for many other applications in bioinformatics [14,15,16]. 

The advantage of using ML is that it can provide a fast, accurate, and un-biased classification of the Zn concentration and water hardness. Another advantage is that it can provide insights into the data and the importance of individual genes that is of use for biologists, who are trying to discover relationships between genes and diseases, but also the relationships among genes [8].

The aim of this study was to evaluate ML techniques to classify Zn concentrations and water hardness from gene expression with 22 genes from *Daphnia magna* collected using qPCR. Furthermore, an analysis of the selected 22 genes was performed using the feature selection method of χ^2^, and feature ranking using a trained Random Forest (RF) and Shapley values in order to rank the genes that are most optimal for the tasks of Zn level estimation and water hardness prediction.

## 2. Materials and Methods

### 2.1. Machine Learning for Gene Expression

The goal in many ML applications is to use a given dataset to train algorithms that learn to perform a specific task. In supervised learning, where the target outputs are known (input data is labeled), the task could be to classify the input data into discreet categories or predict it into a continuous real value. On the contrary, in unsupervised learning the targets are unknown, but the task can then instead be to find patterns or clusters in the unlabeled input data. Classification and prediction can also be used in an unsupervised (or semi-supervised) setting, but the key factor for achieving high performance is to use the correct ML model with a large, labeled training set in a supervised setting [17]. However, there are several examples in the literature of the use of unsupervised learning in gene expression data, such as hierarchical clustering, self-organizing maps, multidimensional scaling, and several clustering algorithms [18,19,20].

The measure of success for a trained ML model is tested by validating how well it can generalize on new unseen test data. A key factor is to avoid poor generalization and overfitting, where the model performs well on the training data but not on unseen test data. This is influenced by the choice of model and model settings (amount of regularization), pre-processing and normalization procedures, the number of samples, and the number and selection of attributes (genes). Another aspect to keep in mind is to avoid any difference in the distribution between the training data and test data; for example, if biological data are collected in different settings such as using different handlers, experimental parameters, organisms, genomic contexts, or growth conditions [21]. This is because ML models are poor at extrapolating to other factors other than those for which the training data were obtained. Other factors such as microarray platform, reference sample, manipulation of the samples, and intrinsic noise in the measurements have also been described as having an influence on the generalization [8]. 

**Figure 1:**
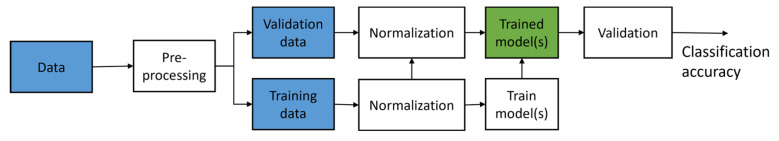
A schematic chart that summarizes the process of training a machine learning model on gene expression data.

In this work, the process of applying ML is to try out several choices for ML model selection, normalization method, feature (gene) selection and use it for training, and then determine which choices give the optimal performance during validation. For validation, a k-fold cross-validation [22] is used where the model is trained using 1/k of the whole dataset as test data and the rest for training data, and then repeat k times with non-repeating samples as the test data. The whole process can be seen in Figure 1.

The code used in this paper can be accessed at https://github.com/martinlangkvist/geneexpression (accessed on 1 May 2023). All models used standard values for hyperparameters from sci-kit learn 1.0.2 with the exceptions that K-nearest neighbor (KNN) used 5 nearest neighbors, Random Forest (RF) used 1000 estimators and max depth of 10, and Neural Network (NN) used ADAM optimizer, learning rate 0.1, 1 layer of 50 hidden units with ReLU activation function.

One key factor of success for any ML model is to have enough training data to capture the distribution of the complex relations between the input and the target output. The amount of data needed depends on the complexity of the data and is difficult to know beforehand, but a common rule of thumb is that the training data should have at least 10 times more samples than the number of input features [23]. For gene expression data, it is generally more costly to acquire more samples than more genes. Another key aspect in ML is feature selection that reduces the data dimensionality, avoids overfitting (to get good generalization), and speeds up the training and inference process. For gene expression data classification, it is, firstly, crucial that the correct genes that correlate to the biological process that is being studied are among the selected genes, and secondly, especially for microarray data, that the redundant genes are removed to avoid noisy input data that reduces the performance of the ML model. A simple unsupervised method for dimensionality reduction is to use the top k number of principal components in a principal component analysis (PCA). However, this method does not guarantee a reduction that optimizes the performance of the predictive model nor give an insight to the biologists about the importance of individual genes. The following section discusses more methods for feature selection for gene ranking.

Finally, one disadvantage of ML predictive models is that they are often black boxes in the sense that the model provides little insight into how it arrived at a certain decision. To achieve good performance on the task the model is trained for, interpretability might not always be necessary. However, if it is achieved, it will result in ML models being more reliable for applications in patient diagnosis, risk assessment, and decision-making. Furthermore, it would give insight into the input data and explain which features are important, which for biologists can provide new knowledge about the importance of certain genes. Some general models for ML interpretability are locally interpretable model-agnostic explanations (LIME) [24], layer-wise relevance propagation [25], and for deep learning model switch post-hoc analyses with feature attribution methods [26,27]. There is ongoing research into how to make ML models more interpretable, specific for gene expression data. The work by [28] utilized a rule-based evolutionary machine learning system for classification of microarray cancer datasets and biologically-constrained architecture [29,30]. The work by [31] used principled pathway attribution methods and game theory in an unsupervised setting to make the learned representations of a deep learning model more interpretable.

### 2.2. Gene Ranking

There are several methods for ranking candidate genes within statistics (hypothesis tests), machine learning techniques (feature selection, model interpretation), and game theory (Shapley values). One common statistical hypothesis test used for gene ranking is the χ^2^-test, which scores each gene by measuring the dependence between each gene and the class so that genes that are independent of class, and therefore irrelevant for classification, get a lower ranking. This has previously been used for gene selection [10].

Within ML there are standard feature selection methods, such as sequential forward selection which has been used for gene selection [32]. Some ML models also have a built-in ability to measure the importance of each feature (gene). For example, a trained Random Forest classifier can compute the feature importance, which takes the sum over the number of splits for each feature across all trees that includes that feature and use it for feature (gene) ranking.

The method for using ideas from game theory for gene expression analysis was proposed [33] where the Shapley value [34,35] was used as a measurement for how much each feature (gene) value contributed to the prediction compared to the average prediction. One advantage over other statistical feature ranking methods, is that the average marginal contribution is calculated over all the possible combinations of genes and for each training example. The calculated Shapley values for each gene can then be used to rank all the genes in terms of the model prediction outcome by (1) the overall classification performance for all classes, (2) the overall classification performance for a specific class, (3) the influence each gene had one a specific sample. Shapley values have previously been used with gene expression data for candidate gene ranking [36,37,38] and gene selection for classification [38]. Other feature selection methods for gene selection that have been used are discretization method [39], support vector machine recursive feature elimination (SVM-RFE) [40], correlation-based feature selection (CFS) [41], and principled attribution [31]. For a general review on feature selection approaches in bioinformatics, please see [42].

### 2.3. Data Collection

The study was conducted using *Daphnia magna*; a freshwater Cladocera commonly used in toxicity testing. The gene expression data used in this study was obtained from our previous study conducted by following OECD guidelines with media modifications to achieve desired Zn and hardness concentrations [43,44]. Exposure groups consisted of daphnids that were subjected to six varying concentrations of Zn (3.1, 5, 10, 25, 50, and 100 μg/L) across three different levels of water hardness: soft (50 mg CaCO_3_/L), medium (100 mg CaCO_3_/L), and hard (200 mg CaCO_3_/L). For details on experimental setup, RNA isolation, and gene expression analysis, see [44].

The data set contains 128 samples with 22 genes and was collected by a single handler. The 22 genes in the data set that is used in this work are: *mta*, *mtb*, *mtc*, *vtg1*, *vtg2*, *ecra*, *ecrb*, *vmo1*, *hsp60*, *ftz1*-*f1*, *e74*, *e75nr*, *usp*, *vasa*, *hsp70*, *hsp90*, *cat*, *gst*, *dap1*, *jhe*, *cyp314*, and *hsp90b*.

## 3. Results and Discussion

### 3.1. Classification and Prediction Results

**Figure 2:**
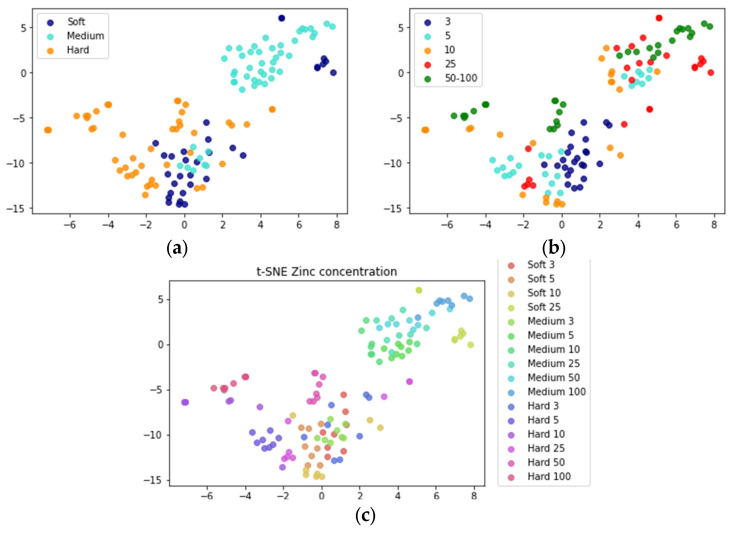
The t-SNE plots for the data set used in this study are colored by (**a**) three water hardness classes, (**b**) five Zn level classes, and (**c**) both water and Zn level.

We first analyzed the dataset by classifying samples for their hardness level, Zn level, and the combination of both. The dataset contained fold change values for 128 samples with 22 genes obtained by using ∆∆Ct method [45]. These genes were selected based on their involvement in key biological pathways that respond to Zn and hardness exposure such as metal regulation, reproduction, and oxidative stress [44]. The data is normalized by removing the mean and scaling to unit variance. Due to the low number of samples, a 10-fold cross-validation was used. t-SNE plots were used to visualize clustering of samples for individual and combined classes (Figure 2). For the hardness class, medium water hardness with Zn levels above 3 μg/L was the most separable from the intertwined soft and hard water with the exception of soft water hardness with 25 μg/L Zn (Figure 2a). There was no clear separation for Zn concentration and combined class (Figure 2b,c). This could indicate that water hardness might be more effective at regulating the gene expression parameter compared to Zn concentrations. However, this could also be explained by changes in hardness levels that were more prominent as well as the number of classes for hardness level that were fewer compared to Zn concentrations.

To further analyze the importance of the classes on gene expression, we then tested eight different classifiers to compare their classification accuracy (Table 1). Classifiers were trained on a small proportion of the dataset and subsequently tested with unlabeled data for class prediction. The best accuracy for hardness classification was achieved by using Quadratic Discriminant Analysis (QDA) with 0.88 ± 0.10 followed by Random Forest (RF) with 0.86 ± 0.12 accuracy. However, QDA resulted in significantly lower accuracy for Zn and combination classification. Neural Network (NN) and RF were the best classifiers, resulting consistent and high accuracy for all classification tested. 

To gain more insight into how each class affected the prediction results and where the confusion between classes occurred, we generated a confusion matrix for class predictions with the best classifiers. The confusion matrix for water hardness class is summarized in Table 2. Machine learning application was able to predict 24 out of 32 (75%) soft water samples correctly while classifying 5 of them as medium and 3 of them as hard water samples. The prediction accuracy was higher for medium water and hard water samples (88% and 90% respectively). 

Similarly, Zn concentration classification prediction for all the tested concentrations ranged between 88% to 92% (Table 3). Interestingly, despite having more classes, the number of confusions for Zn classes were lower compared to hardness classes. 

Finally, we constructed a confusion matrix combining Zn concentration and water hardness classes. The confusion matrix for the combination classifier calculated with Random Forest classifier can be seen in Table 4. Seven of the sixteen classifiers had 100% prediction accuracy. The lowest prediction results were obtained with the lowest Zn concentration in all three hardness groups. The largest confusion is observed for low concentration of Zn in soft and hard water.

### 3.2. Gene Ranking

**Figure 3:**
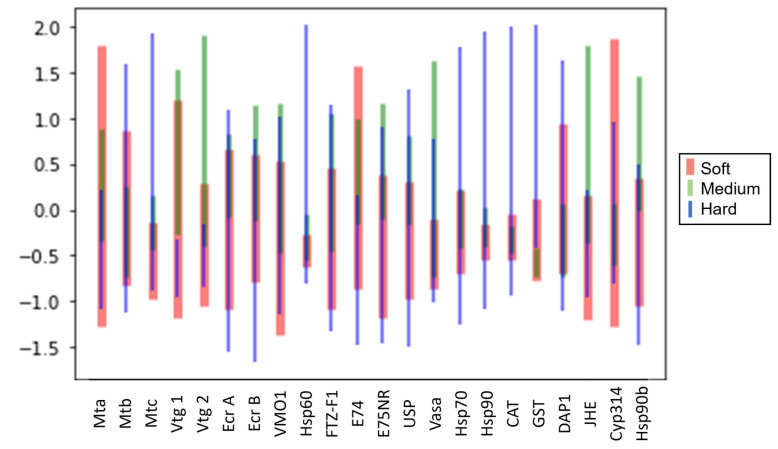
Error bar (mean and standard deviation on normalized raw values) for all 22 genes colored by water hardness.

After investigating the class prediction accuracy, we explored the contribution of each gene to prediction results. Gene analysis is an important step in understanding the biological mechanisms that regulate physiological and biochemical processes in living organisms. The genes were first analyzed by visualizing the error bar for each of the 22 genes in Figure 3. The error bar showed the mean value and standard deviation for the raw fold change values for each gene, and each gene was colored by class. The results showed that 20 out of the 22 tested genes had overlapping values for all three classes of water hardness. However, two genes, *gst* and *vtg1*, did not have overlapping values for medium and hard water hardness (Figure 3). This suggests that the expression levels of these genes could have been influenced by the medium and hard water hardness levels. Interestingly, both genes still overlapped in soft water hardness, indicating that the expression levels of these genes may have been similar under soft water conditions. 

GST, or Glutathione S-transferase, are phase-II detoxification enzymes present in nearly all living organisms which play a crucial role in preserving cellular homeostasis [46]. A separate study revealed that the exposure of Pacu to hard water resulted in an increase in GST activity in both the kidney and gills, as compared to soft water [47]. Another study, using African catfish exposed to different concentrations of Zn, revealed that water hardness had a notable effect on GST levels [48]. Moreover, changes in *gst* expression were also observed in D. magna exposed to copper under varying water hardness levels [49]. On the other hand, vitellogenins (vtg) are major precursors of egg yolk proteins and serve as important biomarkers that reflect changes in reproductive output [50]. Based on our previous study, we observed a phenotypic correlation between the effect of water hardness on *vtg1* levels and reproductive outcomes, whereby both offspring numbers and fecundity for each brood were altered [43]. The hardness effect on reproductive output is also evidenced by numerous studies for *D. magna* [51,52,53,54]. Based on these findings, it could be inferred that the expression levels of most of the analyzed genes were not significantly affected by different water hardness levels, except for *gst* and *vtg1*, which could potentially serve as candidate biomarkers for distinguishing medium and hard water exposures.

In order to test this claim further, we used methods of χ^2^-test, RF feature importance, and Shapley values to generate gene ranking based on their contribution for class prediction in a descending order (Table 5). The Shapley values were calculated using SHAP [27]. Consistent with the previous results obtained by comparing error bars, the *gst* gene has the highest ranking of all three methods, whereas *vtg1* also ranked second for χ^2^-test and third for the other two methods. The results were also similar to previously published data, where effect of the exposure on oxidative stress and reproduction was shown on physiological endpoint levels [44].

**Figure 4:**
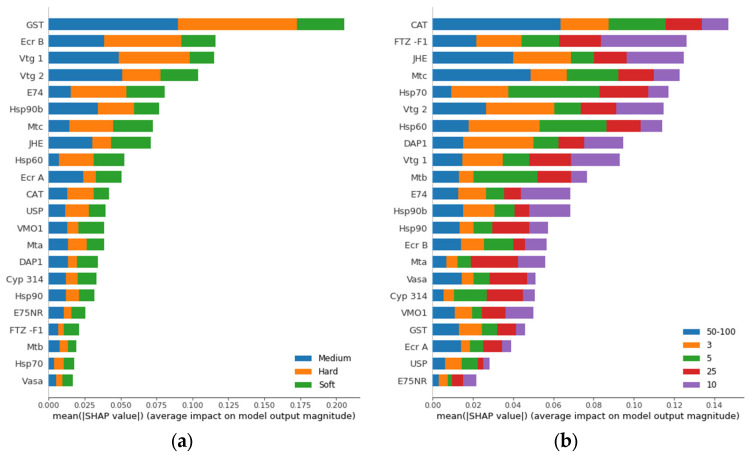
Gene ranking using Shapley values based on contribution to prediction of water hardness (**a**) and Zn concentration (**b**). The genes are ranked by the sum of the total impact for all classes from most total impactful gene (top) to least total impactful gene (bottom) for class prediction.

The advantage of using Shapley values is that the gene importance can be divided per class and per sample. The gene ranking achieved by using overall Shapley values for two classifiers, hardness and Zn concentration, respectively, is shown in Figure 4. The *gst* gene is a strong relevant feature for hardness whereas it does not contribute as much to Zn concentration ranking. On the other hand, *cat* is the most relevant gene for Zn concentration class and has low impact on hardness class. Therefore, gene ranking using Shapley values can be used to identify candidate biomarker genes for each setup.

**Figure 5:**
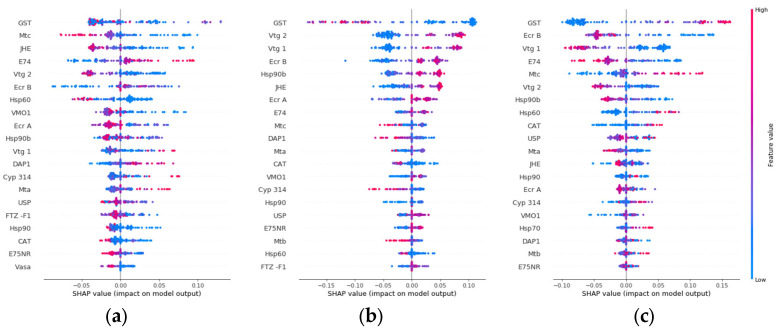
Gene impact on model output for class (**a**) soft, (**b**) medium, and (**c**) hard water hardness using SHAP value impact on model output. The genes are ranked for each class from most impactful gene (top) to least impactful gene (bottom) for class prediction.

The individual gene impact on the model output for each hardness class was further analyzed (Figure 5). According to Figure 5b, when feature values for *gst* are low, the probability of the sample having medium water hardness increases, while high feature values decrease this probability. On the other hand, Figure 5c shows the opposite behavior for hard water hardness, which partly explains why *gst* is the top-ranked contributor for class separation. While *gst* has the overall largest impact for all three classes, other highly ranked genes, such as *e74* and *jhe*, had a low impact on medium and hard classes, respectively. When selecting candidate genes as biomarkers, there is thus an option to select the genes that have an overall impact on all three classes or select genes for a specific class.

**Figure 6:**
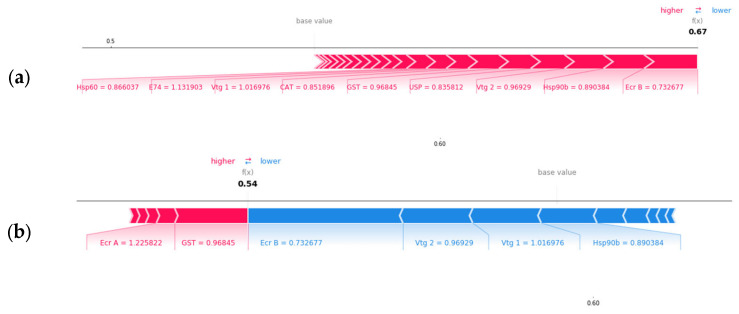

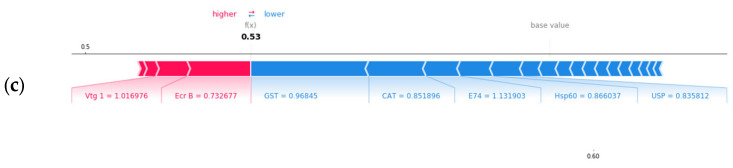
Gene impact on model output for class soft (**a**), medium (**b**), and hard water (**c**) prediction for a soft water sample, respectively. Machine learning predicted the sample class correctly as soft class by assigning highest probability (0.67) to soft water.

Shapley values also allow us to analyze each gene’s contribution to the model output for each class prediction. One example of each gene’s impact on the model output for the soft water class is shown in Figure 6. It was observed that most genes push the probability of the sample being classified as soft from the base value to 0.67 with *e74* being the largest contributor for this sample (Figure 6a). As shown in Figure 6b,c, some genes increase the probability of correct classification, while other genes reduce the class probability from the base values to 0.54 and 0.53, respectively. Therefore, the model correctly predicted this sample as soft hardness since it resulted the highest probability of 0.67 for soft class.

## 4. Conclusions

In this work, eight different standard supervised machine learning classifiers have been trained on qPCR gene expression data for the task of classifying the Zn concentration (5 classes), water hardness (3 classes), and the combination of both (16 classes). The accuracy ranged between 86% and 91% for the first two tasks and reached 82% for the final task. The best model was between Random Forest and Neural Network depending on the task, which could merely be a consequence of non-exhaustive model parameter search. The added benefit of using Random Forest, besides achieving good classification performance, is that it naturally provides feature importance estimates and requires minimal hyperparameter tuning [55]. The main advantage of using Neural Network is that it can achieve higher performance at the cost of needing additional data and more exhaustive model parameter search for when highest possible classification performance is desired. 

The use of Shapley values to visualize the impact of individual genes on overall classification performance, per-class classification performance, and per-sample gene impact has been explored in this paper. Machine learning applications in biological studies should aim to identify previously unknown genes for prediction and/or classification tasks. However, any approach may provide a matching result from a machine learning standpoint and needs to be validated with true results. Therefore, this study constitutes the first step of validation of tested applications on a smaller dataset, where the interpretation of biological significance is established. Our analysis has identified two crucial genes that play a role in oxidative stress and reproduction. The impact of the exposure on oxidative stress and reproduction was evident through phenotypical endpoints, and our gene ranking results were consistent with well-established stress biomarkers for *Daphnia*, as supported by previous studies. This approach of utilizing game theory techniques can enable biologists to manually examine the influence of each gene in different scenarios of interest, rather than relying on only the average gene performance as is typically done in other gene ranking methods.

This work shows that unbiased interpretation of gene expression data by machine learning is a useful tool for toxicogenomics and the work can be extended to other applications of gene expression analysis. Unique gene expression profiles obtained from different exposure conditions can be used to train machine learning, which in turn can be used to predict exposure conditions. The learning outcomes of this study can be further tested with larger data such as transcriptomics, which would potentially lead to the discovery of novel biomarkers.

## Figures and Tables

**Table 1:** Classification accuracy and standard deviation for classifying three classes for multiple classifiers with a five-fold cross-validation.

Classifier	Water Hardness	Zn Level	Combined
Support Vector Machine (SVM)	0.85 ± 0.12	0.82 ± 0.12	0.69 ± 0.06
K-nearest neighbor (KNN)	0.84 ± 0.08	0.78 ± 0.08	0.74 ± 0.08
Random Forest (RF)	0.86 ± 0.12	0.87 ± 0.08	0.82 ± 0.04
Neural Network (NN)	0.85 ± 0.12	0.91 ± 0.09	0.81 ± 0.10
Gaussian Process (GP)	0.81 ± 0.13	0.25 ± 0.03	0.07 ± 0.03
AdaBoost	0.73 ± 0.12	0.37 ± 0.12	0.12 ± 0.00
Naive Bayes (NB)	0.79 ± 0.10	0.60 ± 0.10	0.74 ± 0.08
Quadratic Discriminant Analysis (QDA)	0.88 ± 0.10	0.25 ± 0.03	0.17 ± 0.07

**Table 2:** Confusion matrix for classifying three levels of water hardness for 128 samples using a five-fold cross-validation.

		Predicted		
		Soft	Medium	Hard
	**Soft**	24	5	3
**True**	**Medium**	4	42	2
	**Hard**	3	2	43

**Table 3:** Classification accuracy and standard deviation for classifying water hardness and Zn concentration (16 classes) for multiple classifiers with a five-fold cross-validation.

		Predicted				
		3	5	10	25	>50
	**3**	22	0	1	0	1
	**5**	1	22	0	1	0
**True**	**10**	0	1	21	1	1
	**25**	2	0	0	22	0
	**>50**	0	2	0	1	29

**Table 4:** Classification accuracy and standard deviation for classifying water hardness and Zn concentration (16 classes) for multiple classifiers with a five-fold cross-validation.

		Soft				Medium						Hard					
		3	5	10	25	3	5	10	25	50	100	3	5	10	25	50	100
**Soft**	**3**	0	0	0	0	7	0	0	0	0	0	1	0	0	0	0	0
	**5**	0	8	0	0	0	0	0	0	0	0	0	0	0	0	0	0
	**10**	0	0	8	0	0	0	0	0	0	0	0	0	0	0	0	0
	**25**	0	0	0	7	0	0	0	0	0	0	1	0	0	0	0	0
**Medium**	**3**	4	0	0	0	4	0	0	0	0	0	0	0	0	0	0	0
	**5**	0	0	0	0	0	8	0	0	0	0	0	0	0	0	0	0
	**10**	0	0	0	0	0	0	7	1	0	0	0	0	0	0	0	0
	**25**	0	0	0	0	0	0	0	8	0	0	0	0	0	0	0	0
	**50**	0	0	0	0	0	0	0	0	8	0	0	0	0	0	0	0
	**100**	0	0	0	0	0	0	0	0	0	8	0	0	0	0	0	0
**Hard**	**3**	3	0	0	0	2	0	0	0	0	0	3	0	0	0	0	0
	**5**	0	0	0	0	0	0	0	0	0	0	0	8	0	0	0	0
	**10**	0	0	1	0	0	0	0	0	0	0	0	0	6	1	0	0
	**25**	0	0	0	0	0	0	0	0	0	0	1	0	0	7	0	0
	**50**	0	0	0	0	0	0	0	0	0	0	0	1	0	0	7	0
	**100**	0	0	0	0	0	0	0	0	0	0	0	0	0	0	1	7

**Table 5:** Gene ranking (top has higher importance) using three different ranking methods.

χ^2^	RF Ranking	Shapley Values
*gst*	*gst*	*gst*
*vtg1*	*ecrb*	*ecrb*
*vtg2*	*vtg1*	*vtg1*
*hsp60*	*vtg2*	*vtg2*
*jhe*	*mtc*	*e74*
*hsp90b*	*e74*	*hsp90b*
*cat*	*hsp90b*	*mtc*
*e74*	*jhe*	*jhe*
*hsp90*	*cat*	*hsp60*
*vasa*	*mta*	*ecra*
*mtc*	*hsp60*	*cat*
*ecrb*	*cyp314*	*usp*
*e75nr*	*usp*	*vmo1*
*mta*	*ecra*	*mta*
*vmo1*	*dap1*	*dap1*
*dap1*	*vmo1*	*cyp314*
*cyp314*	*hsp90*	*hsp90*
*ecra*	*mtb*	*e75nr*
*usp*	*e75nr*	*ftz-f1*
*ftz-f1*	*ftz-f1*	*mtb*
*hsp70*	*hsp70*	*hsp70*
*mtb*	*vasa*	*vasa*

## Data Availability

All data supporting the results of this research are included within the article.
